# Remediation of Chromium-Contaminated Soil Based on *Bacillus cereus* WHX-1 Immobilized on Biochar: Cr(VI) Transformation and Functional Microbial Enrichment

**DOI:** 10.3389/fmicb.2021.641913

**Published:** 2021-03-25

**Authors:** Youyuan Chen, Haixia Wu, Ping Sun, Jiaxin Liu, Shixuan Qiao, Dakuan Zhang, Zhiming Zhang

**Affiliations:** ^1^College of Environmental Science and Engineering, Ocean University of China, Qingdao, China; ^2^Key Lab of Marine Environment and Ecology, Ministry of Education, Ocean University of China, Qingdao, China; ^3^Shandong Provincial Key Laboratory of Marine Environment and Geological Engineering, Ocean University of China, Qingdao, China

**Keywords:** Cr(VI)-reducing bacteria, biochar, immobilization, Cr(VI) transformation, stress remission

## Abstract

Microorganisms are applied to remediate chromium (Cr)-contaminated soil extensively. Nevertheless, the microbial loss and growth inhibition in the soil environment restrain the application of this technology. In this study, a Cr(VI)-reducing strain named *Bacillus cereus* WHX-1 was screened, and the microbial aggregates system was established via immobilizing the strain on *Enteromorpha prolifera* biochar to enhance the Cr(VI)-reducing activity of this strain. The mechanism of the system on Cr(VI) transformation in Cr-contaminated soil was illuminated. Pot experiments indicated that the microbial aggregates system improved the physicochemical characteristics of Cr-contaminated soil obviously by increasing organic carbon content and cation exchange capacity, as well as decreasing redox potential and bulk density of soil. Moreover, 94.22% of Cr(VI) was transformed into Cr(III) in the pot, and the content of residue fraction Cr increased by 63.38% compared with control check (CK). Correspondingly, the physiological property of *Ryegrass* planted on the Cr-contaminated soil was improved markedly and the main Cr(VI)-reducing microbes, *Bacillus* spp., were enriched in the soil with a relative abundance of 28.43% in the microbial aggregates system. Considering more active sites of biochar for microbial aggregation, it was inferred that *B. cereus* WHX-1 could be immobilized by *E. prolifera* biochar, and more Cr(VI) was transformed into residue fraction. Cr stress was decreased and the growth of plants was enhanced. This study would provide a new perspective for Cr-contaminated soil remediation.

## Introduction

Cr is the main heavy metal in polluted soil with two speciation of Cr(VI) and Cr(III). The toxicity of Cr(VI) is about 300 times that of Cr(III). More Cr(VI) could diffuse through biofilm with the form of CrO_4_^2–^ or HCrO_4_^–^, leading to oxidization of biomolecules, and biological activity decreased significantly. Lots of technologies for Cr(VI)-contaminated soil remediation have been implemented (Yin et al., 2019; Xia et al., 2021). Among them, the biological technology was applied extensively for its simple process, low cost, and less secondary pollution. A variety of microorganisms, e.g., *Bacillus*, have been selected for Cr(VI) removal (Zeng et al., 2019). Nevertheless, the microbial loss and growth inhibition in the soil environment limit the performance of this technology. Thus, microbial immobilization technology should be implanted for the sake of reducing microbial loss and improving biodegradation efficiency (Luo et al., 2015; Tan et al., 2020b).

The choice of carrier material is an important factor for microbial fixation (Rikmann et al., 2016), and activated carbon, peat, sodium alginate, and biochar are common carriers as described by previous studies (Luo et al., 2015; Abu Talha et al., 2018). Among them, biochar is applied extensively for its simple preparation, low cost, as well as promotion of microbial attachment, metabolic activities, and tolerance for adverse environments (Lee et al., 2016; Xia et al., 2016; Siedt et al., 2021). Biochar has the ability to improve physicochemical properties of soil, including cation exchange capacity (CEC), organic carbon content (OC), and bulk density. Thus, Cr stress for microbes would be remitted.

Biochar could be produced by several materials (Hale et al., 2015). Regulation of surface characteristics of biochar, including functional groups, elements composition, and other factors were the main bottleneck for the biochar application. The raw materials for the preparation of biochar determined the surface characteristics of biochar. As described by previous studies, when sludge, biogas residue, and pine needles were the main raw materials of biochar, lots of heavy metals, polycyclic aromatics, and free radicals appeared on the surface of biochar and have toxic effects on algae, *Rhizoctonia*, and *Streptomyces* (Yang et al., 2019; Li J. et al., 2019). In comparison, studies of biochar derived from *Chlorella vulgaris* contained higher content of P, K, Mg, and Ca, compared with that derived from lignocellulosic biomass, suggesting the excellent perspective of algae as raw material for biochar (Wang et al., 2013). It would be necessary to investigate the effect of raw materials for the characteristics of biochar. Recent studies indicated that biochar made from algae contained hydroxyl, ketone, phenolic, aldehyde, carboxyl, and other polar functional groups had abundant binding sites for heavy metal adsorption (Lehmann et al., 2011; Nautiyal et al., 2016). Moreover, algal biochar had the advantages of adequate alkalinity and sufficient extractable inorganic nutrients; therefore, the biochar could provide favorable survival conditions for microbes (Abdelhafez et al., 2014; Sun et al., 2016; Yu et al., 2017), Nevertheless, adsorption capacity of the biochar is lower than that of lignin biochar, which limited the application of the biochar (Zhou et al., 2019). As described by Abu Talha et al. (2018), the attachment of Cr(VI)-reducing bacteria could decrease the defects of low adsorption capacity and easy saturation of biochar and improve the reuse capacity of biochar. Therefore, it is necessary to explore the interaction between algal biochar and microbes for the remediation of Cr(VI)-contaminated soil. *Ryegrass* is not only a predominant forage species grown in temperate regions but also a kind of turfgrass grown on dry and salty lands (Wu et al., 2005). According to Chen et al. (2017), *Ryegrass* could survive in the salt-alkali estuary region (salinity 0–20‰, pH 7–9) of Licun River, Qingdao, China. Moreover, it was applied globally for heavy metal bioaccumulation reported in previous studies (Healy et al., 2016; Mitchell et al., 2018).

The main objectives of the paper were to (1) screen Cr(VI)-reducing bacteria from the Cr-contaminated soil and prepare biochar-based Cr-reducing microbial aggregation, (2) document the effect the microbial aggregates for Cr transformation and physicochemical property improvement of Cr-contaminated soil, and (3) illuminate the mechanism of the microbial aggregates for soil remediation in the aspect of Cr fraction and functional microbial enrichment.

## Materials and Methods

### Isolation and Identification of Cr(VI)-Reducing Bacteria

The bacteria were isolated from the Cr-contaminated soil samples from Qingdao Hongxing Chemical Factory, and the main products of this factory were inorganic salt. The dilution plate technique method was applied for the strain isolation. The total Cr [Cr(T)] content of the experimental soil is 1205.06 mg⋅kg^–1^, and the Cr(VI) content is 109.83 mg⋅kg^–1^. Ten grams of the soil sample was transferred to a conical flask containing 100 ml of sterile distilled water and shaken at room temperature for 30 min at 140 rpm. The homogenized soil sample was serially diluted and spread on Luria Bertani (LB) agar plate containing 100 mg⋅L^–1^ Cr(r pla_2_Cr_2_O_7_) and then the culture plates were incubated at 30 ± 2°C for 3–5 days. The isolated strain with the function of Cr(VI) reduction was identified by 16S rRNA gene sequencing and selected for the subsequent studies.

### Biochar Preparation

*Enteromorpha prolifera* was purchased from Ocean University of China Biotechnology Co., Ltd. (Qingdao, China). Biochar samples used in this study were produced by slow pyrolysis at 400ishased 0 min under a limiting oxygen atmosphere. After natural cooling and washing by distilled water, the biochar was placed in oven at 60°C and then dried for preparation.

### The Establishment of Cr-Reduction Microbial Aggregates System

*E. prolifera* biochar (1 g) was added into 50 ml of LB liquid medium and sterilized at 121°C for 20 min, and then 20 ml (10% v/v) of strain samples was inoculated in the mixture and shaken at 140 rpm for 24 h in 30°C for the sake of biofilm formation on the biochar surface. After that, the inoculated mixture was centrifuged at 5000 rpm for 5 min and discarded the supernatant. The precipitate was washed three times with Tris–HCl (pH 9) to remove the unfixed bacteria on the surface. The biochar attached with functional strain was established, and the Cr-reduction microbial aggregates system was named as WBC in this study.

### Pot Experiment

To simulate the effect of adding microbial aggregates on the removal of Cr(VI) and plant growth in the soil, a pot experiment was carried out. Before planting, the strain was isolated from Cr-contaminated soil and cultured in LB liquid medium to static conditions and then centrifuged at 5,000 rpm for 5 min, the supernatant was discarded, and the precipitate was rinsed twice with sterile 0.85% NaCl. To simulate the Cr-contaminated soil, soil used in this experiment was contaminated with K_2_CrO_4_ for a week to obtain a concentration of 50 mg⋅kg^–1^ Cr(VI). The groups with different treatments are shown in Table 1: CK^–^ (without Cr contaminated soil), CK (Cr-contaminated soil), W (bacteria), BC (biochar), W+BC (bacteria and biochar), and WBC (microbial aggregates system). Except for the CK^–^, all other experimental groups had the same Cr(VI) content. Soil moisture was maintained around the maximum water holding capacity by applying irrigation (Table 1) and *Ryegrass* were selected as the representative plants in this study. The *Ryegrass* seeds were washed with H_2_O_2_ (3%, v/v) and then deionized water. Then, the seeds were sown in the pots. After germination, equal numbers of healthy and uniform seedlings were kept in each pot. The pots were kept in the greenhouse with a day/night photoperiod of 14/10 h (10,000 lux) and a temperature of 28/22°C (Li J. et al., 2019). After 30 days of culture, the characteristics of Cr in the soil were analyzed. Furthermore, physicochemical properties of soil [pH, oxidation–reduction potential (ORP), CEC, OC, and bulk density], plant growth, and microbial community in the soil were analyzed.

**TABLE 1:** Pot experiment soil treatment.

Group	Cr(VI) (mg⋅kg^–1^)	Bacteria (ml)	Biochar (g)	Inocula (g)
CK-	0.00	–	–	–
CK	50.00	–	–	–
W	50.00	20	–	–
BC	50.00	–	20	–
W+BC	50.00	20	20	–
WBC	50.00	–	–	20

### Analysis Methods

#### Measurement of Soil Cr

The Cr(T) content was determined by the spectrophotometric method and the Cr(VI) content was estimated by the diphenylcarbazide (DPC) method. The Cr(III) content in the sample was calculated by subtracting the Cr(VI) content from the Cr(T) content. The qualification of Cr(III) was mainly based on the subtraction method by most researchers (Wu et al., 2016; Xu Z. et al., 2019; Aparicio et al., 2021).

#### Soil and Plant Characteristics

Soil pH was measured with a pH meter. ORP was measured with the electric potential method. CEC was measured with the barium chloride exchange method. Organic carbon content was measured with potassium dichromate spectrophotometry. Bulk density was measured with the cutting ring method.

The bioconcentration factor (BCF), root concentration factor (RCF), and translocation factor (TF) were key characteristics that could explore the influence of the microbial aggregate system on the transportation and accumulation capacity of *Ryegrass*. The BCF, RCF, and TF for Cr(VI) were determined according to a method described by Sidhu et al. (2017). The BCF, RCF, and TF were calculated as follows:

(1) $$\text{BCF}=\frac{C_{p}}{C_{s}}$$

(2) $$\text{RCF}=\frac{C_{r}}{C_{s}}$$

(3) $$\text{TF}=\frac{C_{s}}{C_{r}}$$

where C_*p*_(mg/kg) was the concentration in the stems and shoots, C_*s*_ (mg/kg) was the concentration in the soil, C_*r*_ (mg/kg) was the concentration in the roots.

#### Analysis of Biochar Surface

The surface topographies of biochar were quantified by ImageJ based on the results of SEM according to Tsui et al. (2018). Briefly, a plugin called SurfCharJ was processed to quantify surface characterization before the image type set as 32-bit. Several surface roughness parameters, i.e., arithmetic mean deviation (*R*_*a*_) and root-mean-square deviation (*R*_*q*_), were fitted. The valleys in the granules were visualized by interactive 3D surface plots generated with a plugin compatible with ImageJ (Prathibha et al., 2011; Chen et al., 2021).

The functional groups on the surface of biochar were analyzed by Fourier infrared spectrometer. The *E. prolifera* biochar was mixed with KBr (spectrally pure) powder with a ratio of 1:10, ground with an agate mortar, and then pressed into tablets. Using a Fourier infrared spectrometer (BRUKER TENSOR 27) with a resolution of 4 cm^–1^, a scanning area of 400–4,000 cm^–1^ was used to determine the surface functional groups of the *E. prolifera* biochar.

#### Microbial Community Analysis

The molecular characterization of the isolated bacteria strain was done by 16S rRNA gene sequencing. The nearly full-length 16S rRNA gene was amplified by PCR using universal primers 27F (5′-AGAGTTTGATCCTGGCTCAG-3′) and 1492R (5′-GGTTACCTTGTTACGACTT-3′), and the PCR products were sequenced by RuiBiotech Ltd. (Qingdao, China). The nucleotide sequences of the 16S rRNA gene were compared with available sequences using the Basic Local Alignment Search Tool (BLAST).

The identification of Cr-reducing strain and analysis of microbial community in the soil were operated by 16S rRNA gene sequencing. The genomic DNA of the sample was extracted following the protocol of the Power Soil DNA extraction kit (MO BIO Laboratories Inc.). The total DNA extracted from the sample was used as a template, and the V3–V4 region of the bacterial 16S rRNA was amplified with the primers (338F 5′-ACTCCTACGGGAGGCAGCAG-3′; 806R 5′-GGACTACHVGGGTWTCTAAT-3′). All reactions were carried out in 25 μl (total volume) mixtures containing approximately 25 ng of genomic DNA extract, 12.5 μl of PCR Premix, 2.5 μl of each primer, and PCR-grade water to adjust the whole volume. PCR reaction was performed in a Master cycler gradient thermocycler (Eppendorf, Hamburg, Germany) set under the following conditions: Initial denaturation at 98°C for 30 s; 35 cycles of denaturation at 98°C for 10 s, annealing at 53°C for 30 s, and extension at 72°C for 45 s; and then final extension at 72°C for 10 min. The PCR products of sample are sequencing by the Illumina MiSeq platform (PE300, CA, United States).

### Statistical Analysis

Each experiment was carried out in triplicate. Statistical analysis was performed using the statistical software SPSS version 20.0. The means of three replicates were subjected to one-way analysis of variance using Tukey’s HSD (honest significant difference) test at a significance level of *P* < 0.05.

## Results and Discussion

### Identification of Cr-Reduction Strain

**FIGURE 1:**
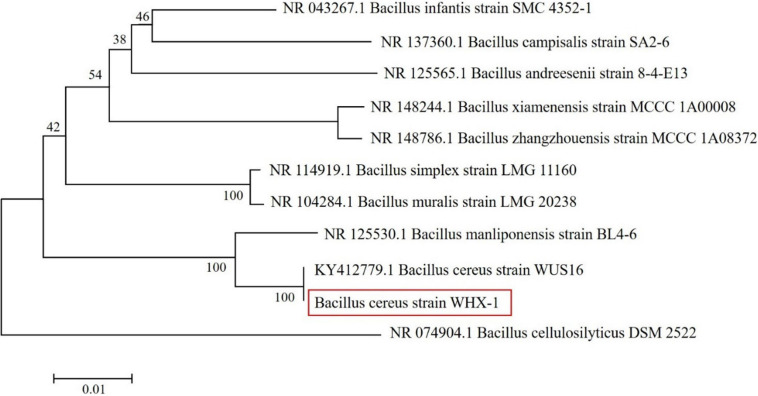
Phylogenetic tree of the strain screened from Cr-contaminated soil.

The strain was isolated from Cr-contaminated soil samples and identified by 16S rDNA genome sequencing. According to the phylogenetic tree, the strain shared close genetic similarity with other identified *Bacillus* spp., especially *Bacillus cereus* strain WUS16 (Figure 1), and the strain was named *Bacillus cereus* WHX-1 in this study. The degrading curve of Cr(VI) by *B. cereus* WHX-1 conformed to first-order kinetics equation and is shown in Supplementary Figure S5.

### Transformation of Cr With Microbial Aggregates Addition in Soil

**FIGURE 2:**
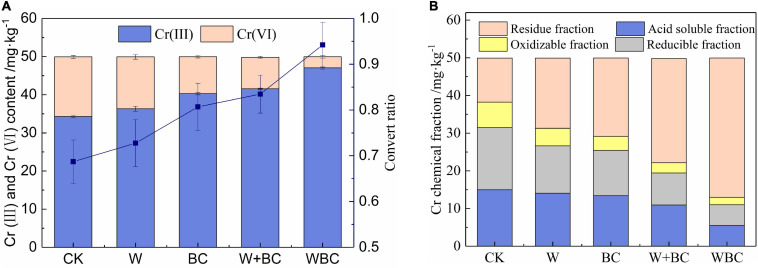
The speciation of Cr in soil with different remediation methods. **(A)** Speciation and convert ratio. **(B)** Chemical fraction.

A pot experiment was conducted to explore the effect of the microbial aggregates containing strain (*B. cereus* WHX-1) on Cr in soil, including the transformation of Cr(VI) and the change of Cr fraction. The soil was full of electron donors, including plant roots and root-related fungi, as well as low-molecular-weight organic acids (LMWOA) produced by humus, rhizosphere bacteria, etc., which have a certain reduction and adsorption effect on Cr(VI). The soil used in this experiment is unsterilized Cr(VI) contaminated soil. The decrease of Cr(VI) concentration may be caused by various factors such as chemistry and biology. LMWOA such as citric acid and tartaric acid contained reduction functional groups, which could be used as a weak reducing agent to reduce Cr(VI) (Xu Z. et al., 2019). In addition, the soil was enriched with bacterial communities, and some species had the ability for Cr(VI) reduction, such as *Oceanobacillus*, *Bacillus*, *Pseudomonas* (Li M. et al., 2019; Zeng et al., 2019; Yao et al., 2020), etc., and Cr(VI) would be reduced directly or indirectly by these microbes. As shown in Figure 2A, the content of Cr(VI) in the group with microbial aggregates addition (WBC) was 2.89 mg⋅kg^–1^, which was the lowest in all treatment, while the soil added with only the strains contained a higher Cr(VI) content of 9.65 mg⋅kg^–1^. The results indicated that WHX-1 could not adapt to the change of soil environment alone during a short process, and the function of the strain could not work fully. Nevertheless, biochar was the common electron donor or electron shuttle agent for Cr(VI) reductions described by Xu X. Y. et al. (2019). When the strains were attached on the biochar, and the microbial aggregates system was established, biochar provides habitat for microorganisms, protects the strain WHX-1 from the stress of environmental factors, and increased the survival rate, leading to uniform distribution of the strain in the soil. Meanwhile, the adsorption of biochar for Cr(VI) in the soil enhanced the interaction between the strain and Cr(VI), and the transformation rate of Cr(C) to Cr(to Crnd the transformatAfter Cr(III) was formed, it was easy to be precipitated or fast combined with the negatively charged organic matter and colloid in the soil, and the mobility is greatly reduced. Studies have shown that cocoa plants could tolerate 100–600 mg/kg of Cr(III). With the increase of Cr(III) concentration, plants alleviate the negative effects through root accumulation and secretion of antioxidant enzymes (Do Nascimento et al., 2018). In addition, pH of most biochar was alkaline and neutral, and the addition of biochar as a bacterial agent carrier could maintain the pH of soil at high level to avoid Cr(III) dissolution, and the mobility of Cr would be restrained (Xu T. et al., 2020).

Cr with different fraction showed varied toxicity for biology, and the effect of the microbial aggregates system on the fraction of Cr in soil is shown in Figure 2B. Compared with CK, the content of residue fraction of Cr in WBC increased sharply with the value of 36.95 mg⋅kg^–1^, while the sum of acid-soluble, oxidizable, and reducible fraction of Cr was lowest with the value of 13.05 mg⋅kg^–1^. As described by previous studies (Chen et al., 2008; Zhang et al., 2017), the bioavailability and eco-toxicity of heavy metals were mainly based on the fraction in soil. The acid-soluble and exchangeable fraction indicated the higher bioavailability of Cr (Yuan et al., 2011), while the oxidizable and residual fraction of Cr were more stable and difficult to accumulate in plants or enriched through food chain; thus, the toxic effect of Cr could be reduced (Tan et al., 2020a). It was concluded that the microbial aggregates system in this study could effectively decrease bioavailability of Cr and enhance the remediation of Cr-contaminated soil.

### Characteristics of Soil Remediated by the Microbial Aggregates System

**FIGURE 3:**
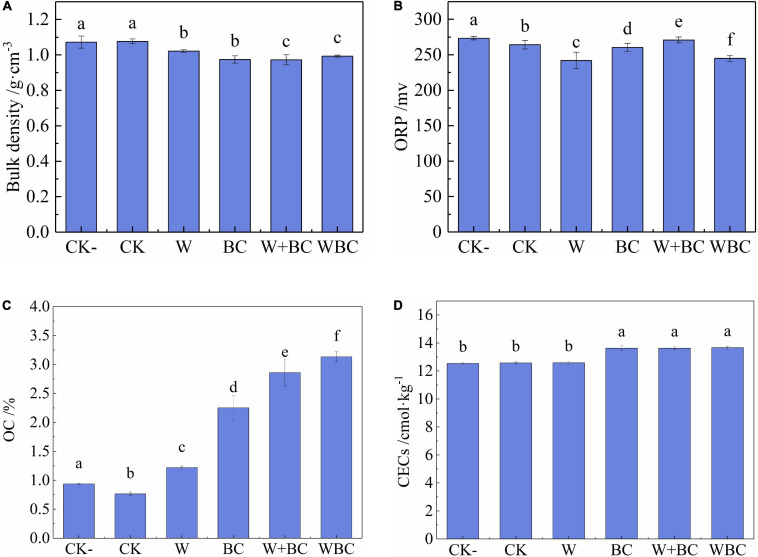
Soil characteristics with different remediation methods. **(A)** Bulk density. **(B)** Redox potential. **(C)** Organic carbon. **(D)** Cation exchange capacity. Error bars represent the standard deviation of the mean (*n* = 3). Values in a given column followed by the same letter are not significantly different (*P* < 0.05) using Tukey test.

The lower content of bioavailable Cr in soil was achieved via the remediation of the microbial aggregates system in this study, and the soil characteristics would be improved correspondingly. The pH of each group was similar at the range of 7.1–7.2; the result did not show significant difference, and the relevant graph can be seen in Supplementary Figure S4. As shown in Figure 3, bulk density and CEC of soil were at the range of 0.97–1.07 g⋅cm^–3^ and 12.53–13.66 cmol⋅kg^–1^, respectively. The two characteristics in each group were similar and did not show significant change. So, bulk density and CEC of soil were not the main characteristics affected by the addition of the microbial aggregates system when compared with OC and ORP according to the results of this study. As described by previous studies, organic carbon could increase nutrient retention and enhance soil biological activity. Soil structure, as well as soil moisture would be improved (Minasny and McBratney, 2018). A significant reduction in ORP might have antioxidant effects, and soil nutrients could be preserved (Manuel, 2009). Moreover, lower soil bulk density and higher CEC indicated porous and fertile characteristics of soil, and plant growth would be enhanced. Soil with microbial aggregates system addition obtained outstanding characteristics, which was suitable to the growth of microorganisms, plants, and other biology.

*Ryegrass* was selected as the representative plant grown in the Cr-contaminated soil, and the effect of the microbial aggregates system on the physiological properties of *Ryegrass* was further studied to evaluate the characteristics of remediated soil for plant growth. As shown in Supplementary Figure S3, Cr content of root and stem of *Ryegrass* in WBC were 0.55 and 4.28 μg⋅g^–1^, respectively, obviously lower than CK and other groups. Furthermore, transfer coefficient (TF) and the biological concentration factor (BCF) were calculated to explore the influence of the microbial aggregate system on the transportation and bioaccumulation of Cr in *Ryegrass*. When the soil was remediated by the microbial aggregates system, TF of Cr in *Ryegrass* in WBC was 0.13, significantly lower than that of CK (0.23) (Table 2). Lower Cr content and TF led to flourishing growth of *Ryegrass*. As shown in Supplementary Figure S2, *Ryegrass* in WBC contained higher content of chlorophyll and dry weight, as well as longer root length and higher plant height. The results indicated that the microbial aggregates could significantly reduce the Cr absorption of *Ryegrass* from the root and the growth of *Ryegrass* was enhanced. By comparison, the soil remediated by the strain without treatment of microbial aggregates formation or only biochar could not recover the soil and the plants to the common states like CK^–^; the main reason could be mainly focused on the effect of biochar for microbial enrichment.

**TABLE 2:** Effect of *Ryegrass* on Cr enrichment coefficient and transfer coefficient under the action of biochar-based Cr-reducing bacteria.

Treatment	Estimators		
	(TF)	(BCF)	Root concentration factor (RCF)
CK	0.23	0.04	0.17
W	0.24	0.04	0.16
BC	0.16	0.02	0.12
W+BC	0.15	0.02	0.11
WBC	0.13	0.01	0.09

### Effects of Microbial Aggregates on the Microbial Community in Soil

**FIGURE 4:**
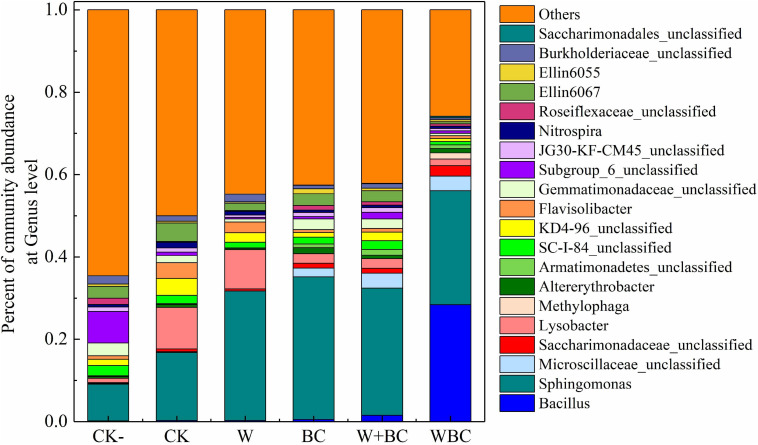
Microbial structure of soil with different remediation methods. Effects of different remediation methods on soil microbial abundance at Genus level.

The addition of microbial aggregates for soil remediation might improve the native microbial structure, and the two types of microorganisms together enhance the remediation of Cr-contaminated soil. As shown in Figure 4, *Bacillus* spp. and *Sphingomonas* spp. were the main microbes of soil in WBC with a relative abundance of 28.4 and 27.7% respectively. Furthermore, *Saccharimonadaceae* unclassified were mainly enriched in the soil of WBC with a relative abundance of 2.5% compared with other groups. The higher abundance of *Bacillus* spp. in soil of WBC indicated that the microbial aggregates formation on the biochar could enhance the inhabitation of exogenous microorganisms in the contaminated soil efficiently; the results were similar to those of Wang et al. (2004). On the other hand, *Saccharimonadaceae* was the main microbe with the function of heavy metal transformation (Zhang et al., 2020) and *Sphingomonas* spp. is the main flocculating bacteria and could strengthen the adhesion of functional microorganisms to biochar (Xu Y. et al., 2020). The enrichment of the two microbes in WBC indicated that the microbial aggregated system could enhance the retention of other functional microbes when *Bacillus* spp. were totally enriched in the system. Moreover, in the soil added with only biochar, the abundance of *Sphingomonas* spp. enriched more with a relevant abundance of 34.6%, indicating the important role of biochar for functional microbe retention in the microbial aggregates system (WBC).

### Remediation Mechanism of Microbial Aggregates

**FIGURE 5:**
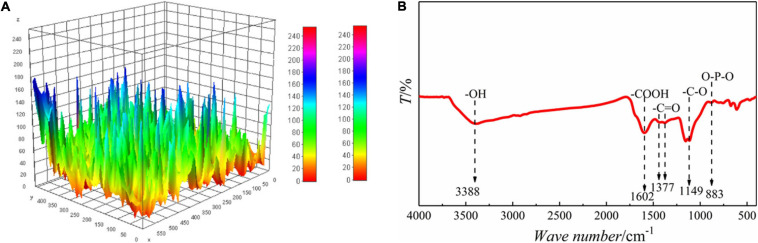
Characteristics of biochar surface. **(A)** 3D Image of biochar surface. **(B)** FT-IR spectra.

*E. prolifera* biochar was selected as the carrier for the immobilization of microbes. As shown in Figure 5, the biochar surface was fitted by SEM combined with ImageJ; the *R*_*a*_ and *R*_*q*_ of biochar was 25.23 and 32.83, and the specific surface area of the biochar was 152.57 m^2^⋅g^–1^. By comparison, the specific surface area of the biochar made from peanut shell at 350°C was only 10.1 m^2^⋅g^–1^ (Xu X. Y. et al., 2019), indicating a rougher and more porous surface of the biochar in this study. Moreover, lots of functional groups, i.e., -COOH (1600 cm^–1^), O-P-O (900 cm^–1^), and amide I (1,600 cm^–1^), were observed on the surface of biochar by FT-IR (Chen et al., 2011). As described by Sheng et al. (2010), these functional groups could contribute to the adhesion of bacteria to solid surface and the microbial aggregate process could be augmented. Moreover, the O-containing functional groups, e.g., -C-O (1,250 cm^–1^) and -C = O (1,400 cm^–1^) (Sheng et al., 2013; Jia et al., 2017), were located on the surface of the biochar, and played as the electron donor moieties of biochar for the Cr(VI) reduction (Xu X. Y. et al., 2019). These abundant functional groups on the biochar surface could enhance the aggregation of functional groups, and the rough and porous surface would provide more active sites for the inhabitation of microbes.

**FIGURE 6:**
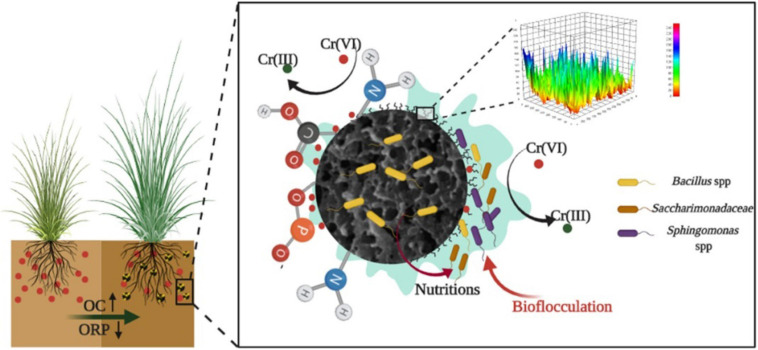
Remediation mechanism of biochar-based Cr-reducing bacteria on Cr-contaminated soil plants.

As shown in Figure 6, biochar could provide nutrient elements to microorganisms except for enhancing microbial aggregation on the surface area. The stable organic carbon fraction of biochar would directly increase the OC and alter the organic components in soil through physical mixing (Han et al., 2020), and porous structures of biochar can decrease soil density (Guo et al., 2020). The environment suitable for the growth of microbes was established. The functional strain was retained by biochar in the microbial aggregates system in this study, and other microbes with the function of heavy metal transformation and bioflocculation were further enriched in the system. The stable microbial system was formed in the Cr-contaminated soil. Finally, the Cr(VI) reduction process was enhanced by the stable microbial aggregates system and the bioavailability of Cr decreased. The stress of Cr for biology in plants was remitted and high performance of the soil remediation system was finally achieved.

## Conclusion

A Cr(VI)-reducing strain named *B. cereus* WHX-1 was screened from Cr-contaminated soil, and *E. prolifera* biochar was selected as a carrier to establish the microbial aggregates system with the strain. The microbial aggregates system could improve the physicochemical characteristics of Cr-contaminated soil obviously by increasing OC and CEC, as well as decreasing redox potential and bulk density of soil. More Cr(VI) was transformed to Cr(III) with residue speciation. High-throughput sequencing indicated that *B. cereus* WHX-1 could be immobilized by *E. prolifera* biochar obviously and Cr-contaminated soil would be remediated and obviously added with the microbial aggregates system. This study would provide a new perspective for Cr-contaminated soil remediation.

## Data Availability Statement

The raw data supporting the conclusions of this article will be made available by the authors, without undue reservation.

## Author Contributions

All authors listed have made a substantial, direct and intellectual contribution to the work, and approved it for publication.

## Conflict of Interest

The authors declare that the research was conducted in the absence of any commercial or financial relationships that could be construed as a potential conflict of interest.

## Supplementary Material

Supplementary Figure 1 Growth of Ryegrass with different remediation methods after 30 days.

Supplementary Figure 2 The physiological characteristics of Ryegrass with different remediation methods. **(A)** Chlorophyll. **(B)** Root length. **(C)** Plant height. **(D)** Plant weight and ratio of dry weight and fresh weight.

Supplementary Figure 3 Cr content in Ryegrass with different remediation methods.

Supplementary Figure 4 Soil pH with different remediation methods.

Supplementary Figure 5 The degrading curve of Cr(VI) by Bacillus cereus WHX-1.
