# Supplementary figures and images for: Remediation of Chromium-Contaminated Soil Based on Bacillus cereus WHX-1 Immobilized on Biochar: Cr(VI) Transformation and Functional Microbial Enrichment
Source: Front Microbiol. 2021 Mar 25;12:641913. doi: 10.3389/fmicb.2021.641913 (PMC8027096; doi:10.3389/fmicb.2021.641913)

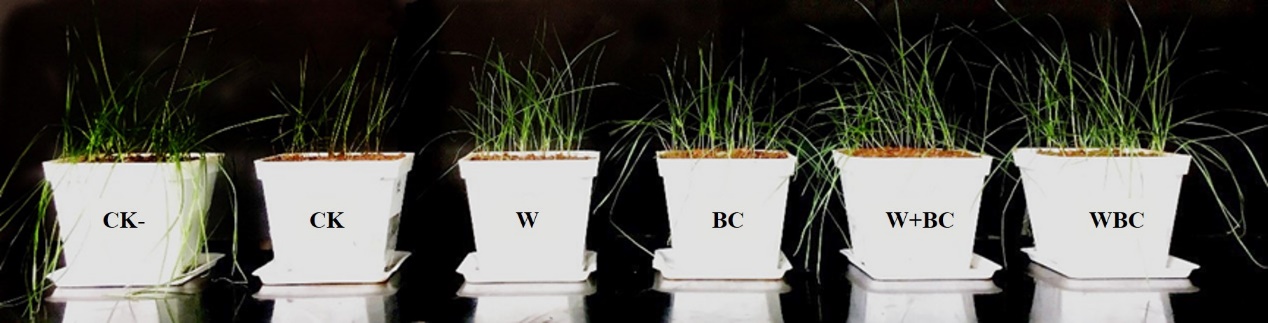

Supplement: Supplementary Figure 1 — Growth of Ryegrass with different remediation methods after 30 days. [file Image_1.jpg]
